# Improvements in elbow motion after resection of heterotopic bone: a systematic review

**DOI:** 10.1007/s11751-014-0192-0

**Published:** 2014-06-17

**Authors:** Ewout S. Veltman, Anneluuk L. C. Lindenhovius, Peter Kloen

**Affiliations:** Department of Orthopedic Surgery, Secretariaat Orthopedie, G4-221, Academic Medical Center Amsterdam, Meibergdreef 9, P.O. Box 22660, 1100 DD Amsterdam, The Netherlands

**Keywords:** Heterotopic ossification, Elbow ankylosis, Systematic review, Burn, Brain injury, Elbow trauma

## Abstract

Complex elbow trauma, severe burn, or a closed head injury render patients at risk for developing heterotopic ossification around the elbow. When heterotopic ossification restricts elbow motion, some patients request surgical resection. We performed a systematic review of the literature to analyze improvement in elbow motion after resection of heterotopic ossification around the elbow. We found that, on average, etiology had little impact on outcome after resection of heterotopic ossification. Resection of heterotopic bone generally leads to improvement of elbow function.

## Introduction

Heterotopic bone formation of the elbow joint has been described to occur with complex fractures and dislocations of the elbow, burns, and closed head injury or coma [1–5]. Although numerous theories have been published, the exact etiopathogenesis of this heterotopic ossification is unclear [6–8].

If elbow motion remains limited in spite of non-operative treatment (i.e., exercises or a static progressive or dynamic splinting program), open or arthroscopic surgical removal of heterotopic ossifications is a widely accepted treatment when a patient requests surgery to regain elbow motion [9, 10]. Recent studies reported comparable improvements in motion after resection of heterotopic bone in ankylosed elbows versus partially restricted elbows [9, 11]. However, surgery remains a technically challenging procedure [11–13].

In this article, we reviewed the range of motion after resection of heterotopic bone around the elbow caused by trauma, burn injury, or head injury. In addition, we have compared the range of motion after resection in patients with ankylosis versus those with partial restriction of elbow motion.

## Materials and methods

We performed a search of all studies on resection of heterotopic bone around the elbow. A search term with Boolean operators was constructed (surgery OR surgical OR operative* OR treatment) AND (heterotopic ossification OR ankylosis) AND (elbow). Two databases (Pubmed/Medline and EMBASE) were searched covering the period from 1978 to 2013. The references of retrieved publications were manually checked for additional studies that would potentially meet the eligibility criteria and that had not been found by the electronic search. All articles describing surgical resection of heterotopic bone at the elbow, written in the English, German, French, or Dutch language, including human adult patients, and with the functional outcome reported were included in this study. Exclusion criteria were case reports with less than five patients per paper, patients with a primary elbow prosthesis, follow-up of <4 year and studies including patients with pre-existing elbow dysfunction.

Abstracts of all articles that were found in the initial search were reviewed independently by two of the authors (EV and AL) for agreement with the eligibility criteria. Then, full texts of selected articles and of those articles with an incomprehensive abstract were read before final inclusion in our review. From each study, we recorded available data regarding years of follow-up, number of patients, baseline patient characteristics, cause of heterotopic bone formation (burn injury, elbow fracture, or head injury/coma), preoperative and postoperative range of elbow motion, postoperative management, additional procedures or medication, and postoperative complications. The data were extracted by one author (EV) and verified by a second (AL).

Improvements in motion are reported for the overall cohort of all studies with a comparison of patients with ankylosis versus those with partial restriction of motion, and for three separate cohorts based on the underlying presumed cause of heterotopic bone (burn injury, elbow fracture, or head injury/coma). All reported averages are sample size weighted.

## Results

**Fig. 1:**
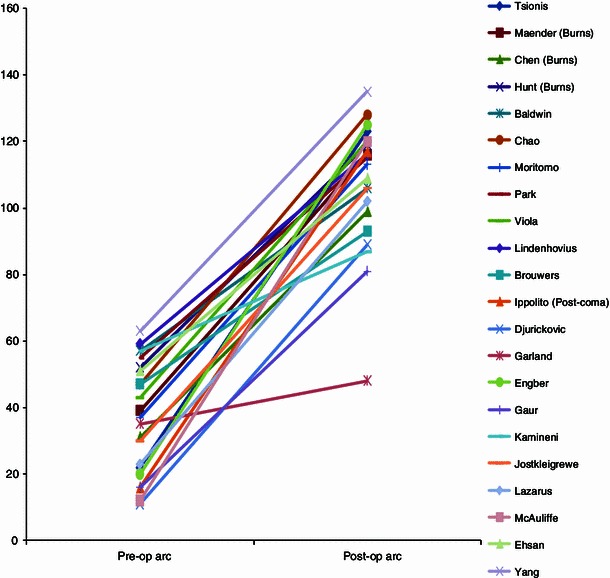
Results of excision of heterotopic ossification for partial ankylosis

**Fig. 2:**
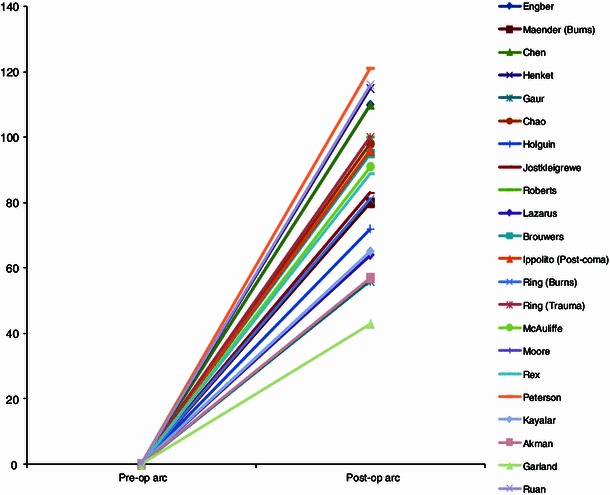
Results of excision of heterotopic ossification for total ankylosis

Thirty-three studies met our eligibility criteria and were included for data analysis [1–4, 12, 14–41]. These studies consisted of two prospective cohort studies and 30 retrospective cohort studies. The results achieved in the individual studies of patients with ankylosis, and patients with partial restriction of motion are visualized in two graphs. Patients with ankylosis of the elbow by definition have a preoperative arc of motion of 0° (Figs. 1, 2). The patients were analyzed based on four cohorts: (1) including all patients, (2) including patients after burn injury, (3) including patients after elbow fractures (including fracture dislocations), and (4) including patients after head injury/coma.

### Cohort 1: all patients

A total of 587 patients (626 elbows) in 33 studies with heterotopic ossification around the elbow were included in the review. Heterotopic bone formation associated with burn injury in 174 elbows described in 13 studies [2, 4, 15, 17, 19, 21, 23–25, 32, 34, 36, 38], it was post-traumatic in 343 elbows reported in 17 studies [1, 12, 14–16, 18, 22, 26, 27, 29, 30, 32, 33, 35, 36, 39, 40], and HO followed head injury in 109 elbows described in nine studies [1, 3, 12, 15, 20, 28, 30, 31, 37].

The average age was 40 years (range 9–76 years), and sixty-eight percent of patients were male. The average time from injury until surgery was 15 months (range 5–123 months). The average follow-up of all patients was 31 months (range 12–80 months).

The average preoperative range of motion of all 626 elbows was 29° (range 0°–65°) with an average 60° of flexion (range 0°–108°) and an average 31° of flexion contracture (range 0°–72°). The average improvement was 67° (range 13°–131°) to an average postoperative arc of motion of 96° (range 43°–131°) with 119° of flexion (range 67°–143°) and 23° of flexion contracture (range 2°–52°).

One-hundred and ninety-three patients had an ankylosis of the elbow [2–4, 14–16, 19–23, 25, 27, 28, 30, 31, 34–37, 39]., whereas 433 patients had a partial restriction of motion [1–4, 12, 15–21, 24–26, 28–30, 32, 33, 35, 38, 40] with an average preoperative range of motion of 44° (range 11°–65°) with 91° of flexion (range 65°–108°) and a flexion contracture of 47° (range 26°–72°).

**Fig. 3:**
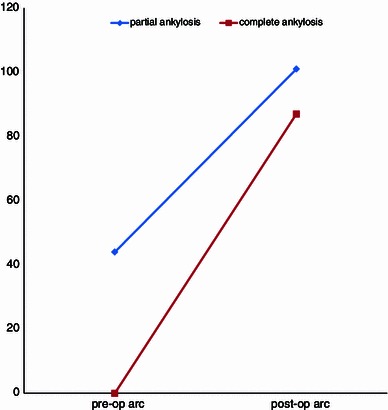
Results for excision of heterotopic ossification in patients after total ankylosis versus partial ankylosis

The improvement in motion in patients with an ankylosis averaged 87° (range 43°–131°), with an average flexion of 115° (range 67°–139°) and an average flexion contracture of 28° (range 5°–45°). Patients with a partial restriction of motion, improved with an average 57° (range 13°–108°) to an average postoperative range of 101° (range 48°–128°), with an average flexion of 121° (range 100°–143°), and an average flexion contracture of 20° (range 2°–52°) (Figs. 1, 2, 3).

### Cohort 2: patients with burn injury

The average time from injury until surgery was 13 months (range 8–17 months) in these 13 studies (148 patients and 174 elbows). The average follow-up time was 40 months (range 15–79 months). Twenty-seven percent of patients (40 patients and 54 elbows) had a complete ankylosis.

**Fig. 4:**
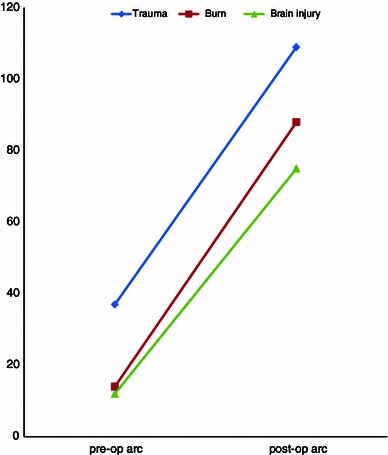
Results for excision of heterotopic ossification in patients after traumatic injury, burn injury, or brain injury/coma

The average preoperative range of motion for all patients with burn injury was 14° (range 0°–52°), with flexion of 43° (range 0°–88°) and flexion contracture of 29° (range 0°–68°). In patients with partial restriction of motion, the preoperative range of motion was 23° (range 11°–52°), with a flexion of 72° (range 65°–88°) and flexion contracture of 49° (range 26°–68°). Mean postoperative range of motion was 88° (range 56°–125°), with a flexion of 119° (range 101°–143°) and extension of 31° (range 15°–45°) (Fig. 4). Range of motion improved with 79° (range 56°–121°) for patients with ankylosis and with 75° (range 65°–105°) for patients with restricted motion of the elbow.

### Cohort 3: patients with elbow fractures

The average time from injury until surgery was 9 months (range 7–123 months) in these 17 studies (341 patients and 343 elbows). The average follow-up time was 30 months (range 12–80 months). Twenty-five percent of these patients had an ankylosis (86 patients and 86 elbows).

The average preoperative range of motion for all patients with elbow fractures was 37° (range 0°–57°), with flexion of 65° (range 0°–108°) and flexion contracture of 28° (range 0°–72°). The average preoperative arc of motion was 52° (range 11°–57°) in patients with partial restriction of motion, with a flexion of 93° (range 74°–108°) and an extension of 40° (range 25°–72°). The average postoperative range of motion was 109° (range 57°–135°), with a flexion of 124° (range 67°–139°) and an extension of 15° (range 0°–35°) (Fig. 4). The range of motion improved with 57° (range 30°–108°) for patients with partial restriction of motion and with 102° (range 57°–131°) for patients with ankylosis.

### Cohort 4: patients with head injury

The average time from injury until surgery was 29 months (range 21–35 months) nine studies (98 patients and 109 elbows). The average follow-up time was 26 months (range 23–31 months). Among patients with heterotopic bone after brain injury, 54 percent had an ankylosis (53 patients and 53 elbows).

The average preoperative range of motion for all patients with brain injury was 12° (range 0°–51°), with flexion of 38° (range 0°–98°) and flexion contracture of 26° (range 0°–68°). The average preoperative arc of motion was 28° (range 16°–51°) in patients with partial restriction of motion, with flexion of 90° (range 81°–98°) and extension of 62° (range 58°–68°). The average postoperative range of motion was 75° (range 43°–117°), with a flexion of 112° (range 85°–137°) and extension of 37° (range 20°–52°) (Fig. 4). Range of motion improved with 49° (range 13°–101°) for patients with partial restriction of motion and with 74° (range 43°–100°) in patients with ankylosis.

### Complications

Wound problems, ulnar neuropathy, recurrence of heterotopic bone, and subsequent surgeries are summarized for those studies that reported these complications.

Wound infection occurred in 19 of the 159 patients (12 %) in which these complications were reported; seven in patients with burn injury (37 %), seven in patients with an elbow fracture (37 %), and five in patients with brain injury (26 %) [1, 17, 20, 28, 33, 39]. Thirty of 266 patients (11 %) had postoperative ulnar nerve dysfunction, which was pre-existent in twenty-four of these patients (80 %); one concerned a patient with burn injury (3 %), twenty-seven were patients with an elbow fracture (90 %), and two were patients with brain injury (7 %) [4, 14–16, 18, 20, 24, 26, 29, 30, 33]. Recurrence of heterotopic ossification occurred in 74 out of 375 patients (20 %); twelve in patients with burn injury (16 %), forty-one in patients with an elbow fracture (55 %), and twenty-one in patients with brain injury (28 %) [1–4, 14, 18, 20, 22–24, 26, 29–33, 36, 38].

Subsequent surgery was performed in 41 out of 299 patients (14 %), for treatment of residual stiffness due to heterotopic bone in thirty-one patients (eight in patients with burn injury and twenty-three in patients with an elbow fracture), for treatment of ulnar nerve dysfunction in two patients (both in patients with burn injury), for infection in five patients (two in patients with burn injury and three in patients with an elbow fracture), for ulnar plate fixation in two patients [one patient with burn injury and one patient with an elbow fracture (2 %)], and for brachial artery rupture in one patient suffering from heterotopic ossification due to brain injury (2 %) [1, 2, 4, 17–19, 22, 28–30, 33, 36, 38].

## Discussion

We studied and compared the results of operative excision of heterotopic ossification around the elbow in patients with various etiologies. We found that the cause of heterotopic bone limiting elbow function had little influence on long-term outcome after surgical contracture release. Trauma patients achieved the best average range of motion (109°) as compared to patients with burn injury (88°) and post-comatose patients (75°). However, when interpreting these numbers, one should keep in mind that 23 of the 343 patients with an elbow fracture (7 %) and 8 of 148 patients (5 %) with burn injury underwent a second procedure for treatment of stiffness due to recurrent heterotopic bone. In patients with heterotopic ossification of the elbow in post-comatose patients, the time between injury and surgery was elongated compared with patients with burn or fracture (29 vs. 39 months, respectively). We are not sure whether this had any effect on final outcome.

Timing of surgery has always been topic of discussion. Classically, surgery is scheduled after maturation of the heterotopic bone; several studies, however, show good results after early excision of heterotopic ossification [5]. Recurrence of heterotopic bone is common in patients with early excision as well as in patients with delayed excision [5]. Subjective patient outcome measures were not widely used in the included studies. Therefore, we feel we cannot make a statement about patient satisfaction following resection of heterotopic ossification around the elbow.

When comparing Fig. 1 with Fig. 2, we noticed the graph shows a difference in inclination for patients with ankylosis, while the graph for patients with partial restriction of motion shows a comparable inclination for most studies. This might reflect a difference in surgeon’s experience with resection of heterotopic bone in the ankylosed elbow. This remains speculative only though as the majority of papers did not report on surgeons’ experience. We have not found another explanation for this difference based on patient characteristics or cause of heterotopic bone. We are not sure whether the extent of heterotopic ossification or the postoperative treatment policy (e.g., the use of non-steroidal anti-inflammatory drugs, irradiation, splinting therapy, or continuous passive motion) has influenced the difference in inclination between the two graphs. Figures 1 and 2 also show less favorable results for the study of Garland et al. [20]. According to the authors of this study, this difference may be caused by the large percentage (nine out of 23 patients, 39 %) of patients with severe physical and/or cognitive impairment included in their study.

The comparison of patients with ankylosis to patients with partial restriction of motion demonstrated a better postoperative range of motion in patients with a partial restriction of motion (101 vs. 87°). However, when we look at the achieved improvements in motion, it was noteworthy that patients with an ankylosed elbow did better than those with a partial restriction (average improvement of 87° in patients with ankylosis vs. an average improvement of 57° in patients with partial restriction of motion). Patients with an ankylosis (who by definition had a range of motion of 0°) gained more increase in motion, measured in absolute degrees, than those with partial restriction of motion. This is consistent with a previously published case–control study [15]. We believe that a possible explanation might be a less extensive surgical approach for patients with partial restriction of motion as compared to patients with ankylosis of the elbow. The data as represented in the literature do not allow conclusions if an improvement from 0° (ankylosis) to 45° is “better” for a patient than an equal improvement in absolute numbers from 45° to 90°. We believe the results in both groups give sufficient benefit to perform surgery in patients that request an operation because of disabling restriction of elbow motion caused by heterotopic ossification.

Resection of heterotopic ossification of the elbow remains a surgical challenge, with a high percentage of complications. Before deciding on performing surgery, both patient and surgeon should be well aware of expected benefits and risks after resection of heterotopic ossification [9, 11].

A weakness of this study is the lack of available level I evidence. The possible flaws of the individual studies are reflected in our conclusions. The reporting of complications was limited in the included studies. Diagnostic imaging techniques, surgical techniques, and insights in optimal timing for resection have changed over the years. Pooling of the overall results was not possible because of the heterogeneity of the data.

A pearl of this study is that we created a comprehensive up-to-date overview of all available literature on the surgical treatment of heterotopic ossification of the elbow. In contrast with other recent studies, we have focused on the difference in long-term outcome for patients with burn injury, traumatic elbow injury, or brain injury and we have compared results between patients with ankylosis of the elbow and patients with partial restricted motion due to heterotopic ossification.

## Conclusions

Currently, the literature lacks high-quality studies as a basis for treatment of heterotopic ossification around the elbow. Even though the incidence of complications is relatively high, we feel excision of heterotopic ossification of the elbow can be a very worthwhile procedure in patients that experience severe functional limitations because of their impaired range of motion. Excision of heterotopic bone can provide a substantial increase in range of motion.
